# Attention Decreases Phase-Amplitude Coupling, Enhancing Stimulus Discriminability in Cortical Area MT

**DOI:** 10.3389/fncir.2015.00082

**Published:** 2015-12-22

**Authors:** Moein Esghaei, Mohammad Reza Daliri, Stefan Treue

**Affiliations:** ^1^Cognitive Neurobiology Laboratory, School of Cognitive Sciences, Institute for Research in Fundamental Sciences (IPM)Tehran, Iran; ^2^Cognitive Neuroscience Laboratory, German Primate Center (DPZ)Goettingen, Germany; ^3^Neuroscience and Neuroengineering Research Laboratory, Department of Biomedical Engineering, School of Electrical Engineering, Iran University of Science and Technology (IUST)Tehran, Iran; ^4^Faculty of Biology and Psychology, Goettingen UniversityGoettingen, Germany; ^5^Bernstein Center for Computational Neuroscience GoettingenGoettingen, Germany

**Keywords:** visual attention, area MT, phase-amplitude coupling (PAC), local field potential (LFP), oscillation, macaque

## Abstract

Local field potentials (LFPs) in cortex reflect synchronous fluctuations in the synaptic activity of local populations of neurons. The power of high frequency (>30 Hz) oscillations in LFPs is locked to the phase of low frequency (<30 Hz) oscillations, an effect known as phase-amplitude coupling (PAC). While PAC has been observed in a variety of cortical regions and animal models, its functional role particularly in primate visual cortex is largely unknown. Here, we document PAC for LFPs recorded from extra-striate area MT of macaque monkeys, an area specialized for the processing of visual motion. We further show that directing spatial attention into the receptive field of MT neurons decreases the coupling between the low frequency phase and high frequency power of LFPs. This attentional suppression of PAC increases neuronal discriminability for attended visual stimuli. Therefore, we hypothesize that visual cortex uses PAC to regulate inter-neuronal correlations and thereby enhances the coding of relevant stimuli.

## Introduction

Local field potentials (LFPs) have been a neural signature of great recent interest. LFPs represent mainly the synaptic activities of local populations of cortical neurons (Buzsáki et al., 2012). For purposes of analysis they are usually divided into different frequency bands to investigate potential functional correlates of each band. Low frequencies (<30 Hz) are thought to represent neural activities on a large spatial scale through which whole populations of neurons are synchronized. High frequencies on the other hand, especially gamma frequencies (30–100 Hz), are generated mainly by local neural activities of small populations of neurons (Buzsáki and Draguhn, 2004). However, high and low frequencies are often coupled (Lisman and Jensen, 2013); an effect called cross-frequency coupling. The main type of cross-frequency coupling links the low frequency (<30 Hz) phase to the high frequency (30–200 Hz) power, known as phase amplitude coupling (PAC) (Canolty and Knight, 2010). PAC has been observed in many cortical regions including sensory areas, hippocampus, and prefrontal cortex of humans (Axmacher et al., 2010), non-human primates (Whittingstall and Logothetis, 2009; Spaak et al., 2012; Wang et al., 2012) and rodents (Chrobak and Buzsáki, 1998; Tort et al., 2009) and it plays key roles in functions such as coding of information in working memory (Raghavachari et al., 2001; Lisman and Jensen, 2013) and motor responses (Yanagisawa et al., 2012).

Furthermore, the power of gamma activity is positively correlated with neural firing rates across different sensory or cognitive states in various brain regions (Liu and Newsome, 2006; Fries et al., 2008; Whittingstall and Logothetis, 2009), but see Ray and Maunsell (2011) for negative correlations. Given this correlation between gamma activity and spike rate, PAC is a potential mechanism by which the low frequency phase of neural activity could determine the firing rate of neurons. Influencing the firing rate of different neurons across time would allow the neural system to utilize PAC to synchronize spike times of different neurons. On the other hand, gamma-band activity is generated by an initial synchronous activity of inhibitory interneurons and sustained by subsequent synchronous entrainment of excitatory neurons (Buzsáki and Wang, 2012). This has been reported to be the case in macaque visual area V4 by Vinck et al. (2013) showing that both putative inhibitory and excitatory neurons are phase-locked to LFP gamma when the behaving animals are visually stimulated. This supports the hypothesis that using PAC, low frequency phase can govern the synchrony of neurons. Consequently, by modulating the magnitude of coupling between gamma activity and low frequency oscillations (PAC power), the neural system could regulate inter-neuronal synchrony. Therefore, we hypothesize that cognitive functions such as selective attention that involve changes in neural synchrony (Ruff and Cohen, 2014), might also involve modulations in PAC power.

Selective attention is an important brain function that enables organisms to selectively process those environmental events that are assumed to be behaviorally significant. Attention can be devoted to different features, objects, or positions and it modulates neural activity (Treue, 2003). Specifically, when primates attend to a stimulus at a given location, the cortical neurons with receptive fields (RF) overlapping that location, fire at a higher rate compared to when the animal attends elsewhere (Reynolds and Chelazzi, 2004). Shifting attention between the inside and outside of a RF has also been reported to modulate the power of LFP at different frequencies. The LFP power at low frequencies (<20 Hz) decreases in extrastriate areas V4 and MT when shifting attention into a RF (Fries et al., 2001, 2008; Khayat et al., 2010). Similarly, the coupling of spikes to the LFP at low frequencies decreases when attending inside the RF in area V4 (Fries et al., 2001, 2008). These findings suggest that neurons become decorrelated when spatial attention is directed to their RF. Along the same lines Mitchell et al. (2009) reported that the synchrony of neurons in low frequencies decreases with attention and Cohen and Maunsell (2009) showed that the inter-neuronal correlations decrease with attention however see Ruff and Cohen (2014) for an opposite observation. Given these findings, we hypothesize that attention may use PAC as a mechanism to control inter-neuronal synchrony, i.e., by modulating PAC power attention could regulate the synchrony between neurons. Therefore, we investigate whether selective attention influences PAC power in LFPs recorded from extrastriate visual cortex of macaque monkeys.

## Materials and methods

### Behavioral paradigm and recording

**Figure 1:**
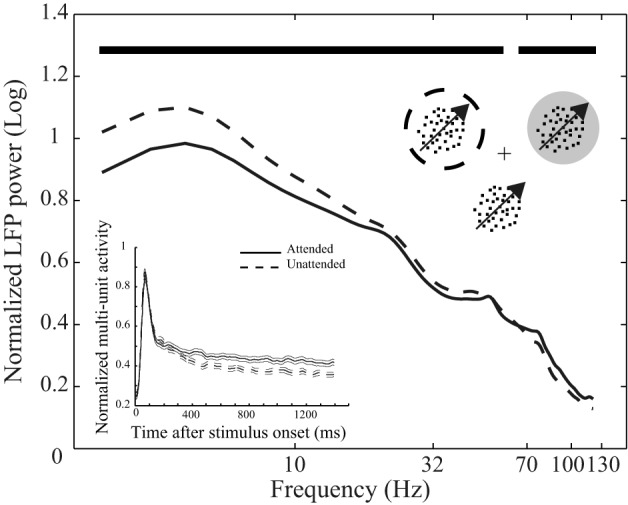
**Behavioral paradigm and attentional modulation of neural activity**. Top inset depicts the behavioral paradigm: Three monkeys were trained to attend to the cued moving random dot pattern (RDP, the target stimulus) among two (monkey H) or three (monkeys C and T) moving RDPs and respond to a change of its direction. The target could be inside or outside the receptive field (RF) of the recorded neuron. The cross represents the fixation point, the dotted circle represents the cued position and the gray circle indicates the RF. The circles are not shown in the experiment. The curves reflect mean LFP power at different frequencies across the 146 recording sites from the three monkeys separated by attention condition. LFPs come from the interval 401–1400 ms after onset of stimuli and are normalized per-site by the average power across frequencies in the two attention conditions. Data are shown on a logarithmic scale and time points with a significant difference in LFP power between the two attention conditions are marked with bold black lines above the curves. The bottom inset similarly represents the average spike density function based on multi-unit activity (MU) in the two attention conditions pooled across the three monkeys. Data are normalized by the maximum value for each recording site and aligned to the stimulus onset. Solid and dashed lines represent neural activity from trials with the target inside and outside the RF, respectively. Error bars show the standard error of the mean (SEM).

Three male monkeys participated in this study. All procedures of this study have been approved by the regional government office (Niedersächsisches Landesamt für Verbraucherschutz und Lebensmittelsicherheit (LAVES)). We trained the animals to direct their attention to one of two or three coherently moving random dot patterns (RDPs) until this target stimulus underwent a brief direction change. At the beginning of each trial the monkeys had to touch a lever and fixate a central fixation point. A cue was then presented indicating the location of the upcoming target stimulus. The cue was a static RDP shown in the same position as the upcoming target for 455 ms (monkey H) or a small rectangle placed on a virtual line connecting the fixation point to the target, for 250 ms (monkeys C and T). After a short blank period, the moving RDPs were presented and the monkey had to release the lever when the target underwent a direction change. The change time was picked from a uniform random distribution 680–4250 ms (monkey H) or 13–4250 ms (monkeys C and T) after the onset of the stimuli. In a given trial all RDPs moved in the same direction, randomly chosen out of eight possible directions (0–360° with steps of 45°) for monkey H and to the preferred or anti-preferred direction of the recorded neuron for monkeys C and T. They were rewarded for releasing the lever within an interval of 150–650 ms (monkey H) or 60–700 ms (monkeys C and T) after the direction change of the target. The direction change could also occur in an un-cued stimulus (distracter). The monkeys had to ignore such changes. A response to these changes would lead to the termination of the trial without reward (Figure 1). Our paradigm entailed two types of trials; trials in which attention was focused inside the RF and trials in which attention was focused outside the RF. Since there is no other difference in terms of the shown stimuli or the behavioral task between the two trial types, the divergence of multi-unit (MU) activities shown in Figure 1 reflects the location of spatial attention, confirming that the monkeys actively attended the target and ignored the distracter (s). Monkeys H, C and T correctly released the lever for direction changes of the target in 86, 71 and 83% of trials in which they did not break their fixation, respectively. The average number of hit trials for a given attentional condition was 63 for animal H, 10 for animal C and 6 for animal T.

MU activity and LFPs were recorded from area MT using a five-electrode recording system (MiniMatrix; Thomas Recording). We recorded from up to all five electrodes (with the impedance of 2 MΩ) simultaneously. In sessions with simultaneous recordings we made sure that the RFs of the different units overlap sufficiently for all to contain the stimulus placed in the RF.

### Data analysis

We carried out our analyses on sessions with at least five correct trials in each attention condition. All analyses were carried out using MATLAB (Mathworks, Natick, MA). The power spectral density (PSD) in Figure 1 was calculated by taking the absolute values of the output of MATLAB's *fft* function applied to the LFPs. Next we replaced those power components corresponding to the 50 Hz line noise as well as the 76 Hz monitor refresh rate noise by the mean power of the two neighboring frequency components. The result was averaged per site across trials from each attention condition, convolved with a Gaussian of σ = 2 and finally normalized to the mean frequency power across frequencies of both attention conditions. In order to smooth MU activity, we convolved spike trains with a Gaussian function (σ = 15 ms) and normalized the result by the maximum value across the two attention conditions for each recording site. The beginning time for divergence of the MU activity between different attention conditions was determined by the first millisecond after 10 consecutive milliseconds with significant difference between the conditions (*p* < 0.01, paired *t*-test).

**Figure 2:**
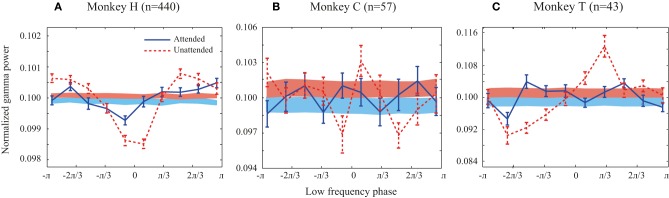
**Probability distribution functions (PDFs) of the high frequency band (30–120 Hz) relative to the phase of the low frequency band (1–8 Hz) for all animals**. **(A–C)** Solid line curves show the PDFs for each attention condition averaged across trials. Shaded bands show the average of PDFs randomly shifted circularly for each site pair. Attended and unattended conditions are shown in solid blue and dashed red curves, respectively. In the shaded bands the color transition line represents the mean of the random PDFs, while the upper and lower bounds reflect SEM for the unattended and attended condition, respectively. Error bars show SEM.

**Figure 3:**
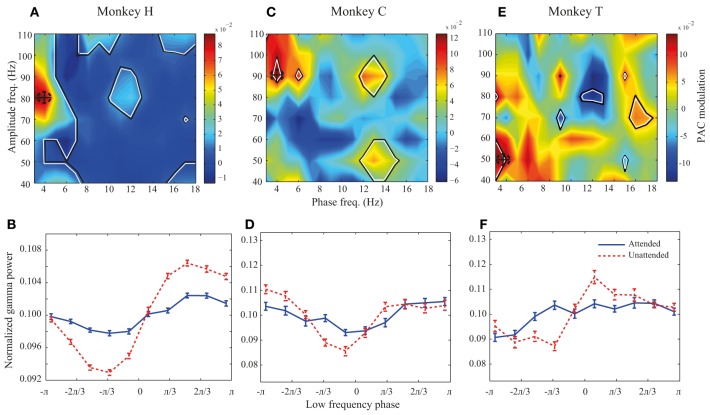
**PAC modulation map for monkeys H, C, and T. (A,C,E)** The heat-maps show the peak-to-peak amplitude for the unattended condition subtracted by the peak-to-peak amplitude for the attended condition across different frequency pairs. Black lines indicate frequency pairs with significant PAC modulation [*p* < 0.05, permutation test; corrected for multiple comparisons (See Materials and methods)]. A dashed circle in each panel shows the frequency pair with the highest attentional modulation of peak-to-peak amplitude. **(B,D,F)** The PDFs for the attended and unattended condition for the frequency pair with the maximum PAC modulation, as indicated by dashed circles in the heat-maps. Error bars show SEM.

LFPs were phase-aligned, accounting for the phase lags of the recording system using the method of Nelson et al. (2008). The LFP for each trial was subtracted by its mean and normalized by its σ. We removed the 50 and 76 Hz noises by subtracting the band-pass filtered components between 48–52 and 73–78 Hz using the EEGLAB toolbox (*eegfilt* function; Delorme and Makeig, 2004). We also used the same routine (with the filter order of 3^*^(sampling_rate/low_cutoff_freq) and assuming each given LFP signal as one epoch) to filter the LFPs into different frequency bands to calculate the probability distribution functions (PDFs) of the high frequency power relative to the low frequency phase (Figure 2). In order to avoid edge effects, created by the cut-off at the beginning and end of an LFP time segment, we appended 300 ms data from the same trial to both ends of the LFP and later removed the corresponding part from the filtered signal. To calculate the PDFs (Figure 2), we band-pass filtered the LFP signal coming from a given trial into low (1–8 Hz) and high frequency (30–120 Hz) components. Next, we used a Hilbert transform to calculate the analytic signal of both components. Instantaneous phases of the low frequency component were quantified by calculating the angles corresponding to the low frequency component's analytic signal. The instantaneous high frequency power was quantified by calculating the second power of the absolute analytic signal based on the high frequency component. The PDF was then computed by calculating the normalized gamma power at each of ten equal phase bins partitioning the phase range (-π, π) (Supplementary Figure 1). PAC power was quantified by calculating the peak-to-peak amplitude of the computed PDF and normalizing it by the mean probability across phase bins (Tort et al., 2010). Permutation tests were used to calculate the significance of separability between mean PDFs across attention conditions,: The PDFs calculated for all recording pairs in the two attention conditions were shuffled and divided into two groups of the same size (10^6^ iterations for monkey H and 10^5^ iterations for monkeys C and T) and for each shuffling iteration the peak-to-peak amplitude difference of the mean PDF across the two groups was calculated. Comparing the real peak-to-peak amplitude difference with the generated population gave us the *p*-value of the separability. To find significant frequency pairs in Figures 3A,C,E we corrected for multiple comparisons using the Bonferroni method.

Before calculating the neural discriminability (*d*′) we ensured that the distribution of firing rates across task conditions (attended vs. unattended and preferred vs. anti-preferred direction) for each recording site did not differ significantly from a normal distribution using the Kolmogorov-Smirnov test (*p* > 0.05). We calculated d′ using the formula:

$$\begin{array}{l} d^{\prime }=\frac{{\text{R}}_{pref}-{\text{R}}_{antipref}}{\sqrt{\frac{{\sigma^{2}}_{pref}+{\sigma^{2}}_{antipref}}{2}}} \end{array}$$

where R denotes the average firing rate when presenting the preferred (pref) or antipreferred (antipref) stimulus and σ^2^ denotes the variance of the firing rate in the two stimulus conditions.

## Results

We trained three monkeys to direct their attention to one of two or three moving random dot patterns (RDPs) until this target stimulus underwent a brief direction change (Figure 1). Figure 1 shows the power of the LFPs between 401 and 1400 ms after stimulus onset at different frequencies for two types of trials in which attention was directed to the stimulus inside (attended) or outside (unattended) the RF of the recorded neuron. Powers are averaged across recording sites of the three monkeys after normalizing per-site by the mean LFP power across different frequencies of the two attention conditions. The two curves clearly show that switching attention into the RF causes a significant decrease in the power of LFP oscillations below 54 Hz (with frequencies < 18 Hz having the maximum modulation relative to mean power) and a significant increase in the power of frequencies above 67 Hz (*p* < 0.01, sign test, corrected for multiple comparisons) (see Materials and methods for details). Similarly, normalized MU activity averaged between recording sites for all monkeys is reflected in the bottom inset for the two conditions. The two curves corresponding to each attention condition diverge 370 ms after the onset of the stimuli (see Materials and methods for details). These findings are in line with previous observations on the attentional modulation of oscillatory neural activity (Fries et al., 2001, 2008; Khayat et al., 2010) and time course of spatial attentional modulation of neural activity in visual cortex (Treue, 2001; Busse et al., 2008; Katzner et al., 2009) and confirm that the monkeys followed the spatial instructions provided by the cue.

We first investigated whether there is a link between low frequency (1–8 Hz) phase and the power of high frequency LFPs (30–120 Hz) during stimulus presentations. Focusing on the sustained rather than the transient part of the neural responses and on frequencies as low as 1 Hz, we analyzed the LFP signals between 401 and 1400 ms after target onset. We only included trials in which the direction change of the target occurred after this time window. The probability distribution function (PDF) of the power of high frequency LFPs (30–120 Hz) relative to the phase of low frequency LFPs (1–8 Hz) was calculated for each trial (for details see Supplementary Figure 1 and Materials and Methods). The low and high frequency LFPs were extracted from the same recording site or from simultaneously recorded sites and the corresponding PDFs were then averaged across trials for each pair of sites in each attention condition. Figure 2 shows the average PDF for the three monkeys averaged across site pairs in the two attention conditions. The PDFs present the average power of high frequency LFPs occurring at each of ten equally wide low frequency phase bins. In order to control if the PDFs of different pairs are consistent in terms of their phase-power dependencies, we randomly shifted each site pair's PDF circularly by a random phase and averaged them per condition for each monkey. The results are shown as shaded curves in Figure 2. Both conditions led to uniform PDFs across the three monkeys. Furthermore, as a simple quantification of the level of dependency between the two oscillation factors we measured the peak-to-peak amplitude of the mean original PDF and the mean random PDF (Supplementary Figure 1). The peak-to-peak amplitude was significantly greater for the original data than the random data in both attention conditions for all monkeys (*p* < 0.05, permutation test) (except for monkey C in the attended condition possibly due to the low number of trials available). This shows that the phase-power relationship is not random across site pairs and that the low frequency phase and high frequency power depend on each other. This relationship suggests that there is a link between the phase of low frequency and the power of high frequency oscillations in LFP during specific cognitive states, consistent with previous findings in different cortical regions (Canolty and Knight, 2010; Lisman and Jensen, 2013). The PDFs of the original data (red solid curves) suggest that the dependency is especially pronounced in the unattended condition. This is particularly apparent for monkey H (Figure 2A), where the peak-to-peak amplitude in the unattended condition is significantly larger than the attended condition (*p* < < 0.001, permutation test) (see Supplementary Figure 2 for an example trial's LFP in attended and unattended condition). Monkeys C and T similarly showed a significant decrease of the peak-to-peak amplitude (as a measure of PAC power) with attention (*p* < 0.01 for both monkeys; permutation test). These results suggest that the power of PAC, defined as the degree of dependence between low frequency phase and high frequency power, decreases with spatial attention.

We further investigated how attention modulates PAC power across different low-high frequency pairs. We calculated the difference between the peak-to-peak amplitude in the two attention conditions for different low-high frequency pairs. All possible combinations of low frequency bands of 4 Hz width with the lower bounds between 1 and 16 Hz (in steps of 1 Hz) and high frequency bands of 20 Hz width with the lower bounds between 30 and 100 Hz (with steps of 10 Hz) were included in calculating this difference. Figure 3A shows the result for monkey H. Colors code the difference between peak-to-peak amplitudes across the two attention conditions, with warm colors marking frequency pairs at which PAC is higher in the unattended condition and cool colors representing regions with higher PAC in the attended condition. The regions marked with black lines represent frequency pairs at which the PAC power modulation is statistically significant. The highest PAC power difference between the two attention conditions occurs at the combination of 2–6 and 70–90 Hz indicated by a dashed circle (Figure 3A). In addition many frequency pairs around this combination are also significantly affected by attention, creating a patch of frequency pairs that show a decreasing effect of attention in their PAC power. The PDFs at this maximum modulation point are plotted in Figure 3B showing a clear difference between the peak-to-peak amplitude of the two attention conditions in this frequency pair (*p* < < 0.001, permutation test). The same analyses were carried out for monkeys C and T (Figures 3C–F). Maps showing the similar modulation of peak-to-peak amplitude normalized to the mean peak-to-peak distance are shown in the supplementary material (Supplementary Figure 3). For monkey C the frequency pair with the highest PAC modulation was found at (2–6 and 80–100 Hz; Figure 3C) and for monkey T the highest PAC modulation occurred at (2–6 and 40–60 Hz; Figure 3E). Similar analyses carried out on another dataset recorded from monkey T (Katzner et al., 2009) show similar results in frequency pairs around 1–5 and 80–100 Hz (Supplementary Figure 4). The PDFs at these frequency pairs are shown in Figures 3D,F correspondingly. For all three monkeys, the trough of the PDFs in the unattended condition occurs within -π/3 – 0 radians which corresponds to the rising phase of a low frequency cycle.

Although PAC is decreased at frequency pairs with maximum modulation for all three monkeys, there are several frequency pairs that conversely show significant increase of PAC when attention shifted toward the RF (for instance frequency pair 4–8 and 50–70 Hz for monkey H; Figure 3A). However, as shown in Supplementary Figure 5, these frequency pairs are rare compared to the frequency pairs with suppressive PAC modulation (*p* < < 0.001, sign test).

It may be argued that the modulation of PAC is a side effect created by the attentional modulation of low frequency power. As shown in Figure 1 in area MT and consistent with reports in other visual areas (Fries et al., 2001, 2008) switching attention into the RF leads to a decrease of oscillation power in low frequency (<20 Hz) LFP bands. It is therefore conceivable that the estimation of the low frequency component is less accurate in the attended compared to the unattended condition (assuming a constant level of noise across the two conditions). To rule this out we conducted similar analyses as in Figure 3 on a selection of trials for monkey H that showed the opposite attentional effect on low frequency power, i.e., trials in which directing attention into the RF caused a higher oscillation power in low frequency (<20 Hz) LFP than trials with attention directed outside RF. The peak modulation of PAC for these trials occurred at the same frequency pair as in Figure 3A (2–6 and 70–90 Hz) with a significant decrease of PAC with attention (*p* < < 0.01, permutation test; Supplementary Figure 6). This suggests that the attentional mechanisms modulating PAC and the low frequency power of LFPs are independent.

One potential advantage of PAC modulation is to regulate the synchrony of neuronal spikes using the link between gamma power and spike rate (Liu and Newsome, 2006; Fries et al., 2008; Whittingstall and Logothetis, 2009). Consequently, we hypothesize that attention harnesses PAC to increase neural discriminability by decorrelating neuronal spikes (Mitchell et al., 2009). To test this hypothesis, we first investigated if there is any link between gamma power and spike rate in each attention condition. Second, we checked for any potential link between PAC power and neural discriminability. The analyses were conducted on data from monkeys H and C while monkey T's data were excluded due to the low number of trials in the different sensory conditions.

To test for any link between gamma power and spike rate in time, we calculated the power of gamma and spike density functions (SDFs) across time for each attention condition. Next, the median gamma power across trials with the same stimulus properties (preferred or anti-preferred stimulus) was calculated and time points were divided into two classes relative to this median. We found that SDF values corresponding to intervals of the class with higher gamma power were significantly larger than those corresponding to lower gamma power (*p* < 0.001 for monkey H and *p* < 0.05 for monkey C; sign test). The gamma band for each of the monkeys was selected according to the frequency pair with the largest PAC modulation, i.e., 70–90 and 80–100 Hz for monkeys H and C, respectively. Furthermore, we quantified the gamma power-SDF link by computing the normalized difference between mean SDF values corresponding to the two gamma power classes [(mean SDF at higher gamma power − mean SDF at lower gamma power)/(mean SDF at higher gamma power + mean SDF at lower gamma power)]. For both monkeys this link was larger in the unattended condition than the attended condition, although it was not statistically significant for monkey C (possibly due to far lower number of recording sites compared to monkey H; Monkey H: *p* < 0.001 0.005 ± 0.034(σ) (attended) and 0.025 ± 0.04(σ) (unattended), Monkey C: *p* = 0.18 0.017 ± 0.054(σ) (attended) and 0.056 ± 0.05(σ) (unattended); sign test).

Next, we investigated the potential link between PAC power and neural discriminability. We computed neural discriminability (based on responses to the preferred and anti-preferred stimulus direction) by computing *d*′ for each recording site (see Materials and methods for details). We then focused on recording sites with an increase of neural discriminability when attention was shifted into the RF. This is in line with previous reports of an increase of neural discriminability measures, such as the Fano factor and tuning curve height with spatial attention (McAdams and Maunsell, 1999; Mitchell et al., 2009 among others). PAC analyses were similarly focused on site pairs were the high frequency component came from sites with a positive attentional modulation of neural discrimination. We divided the population of site pairs into two same-sized groups where the difference between the mean PAC power at the frequency pair with highest modulation (Figures 3A,C) across the two groups was largest (in 1000 random independent divisions). Next, the mean neural discrimination for the gamma-providing sites of each site-pair group was calculated and the two values were compared one hundred times. The mean neural discrimination corresponding to the site-pair groups with smaller PAC power was significantly larger than the groups with higher PAC power in both monkeys H and C (*p* < < 0.001 for both monkeys; sign test). This, together with the observation that gamma power is linked to MU spike rate (especially in the unattended condition), suggests that a reduction in PAC power increases neural discrimination.

## Discussion

This study investigated the coupling between the phase of low frequency (1–8 Hz) oscillations and the power of high frequency oscillations (30–120 Hz) in LFPs of area MT in macaque visual cortex and the influence of spatial attention on this coupling.

Our data show that in MT the power of high frequency LFPs is locked to the phase of low frequency LFPs. We further found that spatial attention modulates the coupling between the low frequency phase and high frequency power; shifting attention to the RF decreases PAC power for frequency pairs with the phase-providing frequency below 7 Hz. Our observation of PAC in MT is consistent with reports from a variety of cortical areas in humans, non-human primates and rodents (Canolty et al., 2006; Jensen and Colgin, 2007; Buzsáki and Diba, 2010; Lisman and Jensen, 2013). It is further consistent with Bosman et al. (2012)'s observation of coupling between the theta phase in V4 and the V1–V4 gamma coherence in an attention task.

The previous observation that spatial attention modulates the power of LFPs at different frequencies (Fries et al., 2008; Esghaei and Daliri, 2014) indicates that the effect of attention on PAC may be a side effect of an attentional modulation of LFP power. To rule this out, we generated random signals with the same spectral power properties as the original LFPs for trials of both attention conditions. The generated dataset matched the original dataset in terms of the number of recordings, trials and trial lengths. The randomly generated dataset of signals however did not show a statistically significant PAC difference between the two attention conditions. This confirms that the attentional modulation of PAC is not a side effect of power spectrum modulations of LFPs by attention.

Bosman et al. (2009) found that a low frequency oscillation of ~3.3 Hz dominates microssacade occurrence and that microssacade-triggered LFPs contain consistent low frequency as well as gamma-band components. Given this observation, it may be argued that the decreasing effect of attention on PAC power is caused by the dissociation of low frequency component to microssacade onset in attended condition. This would consequently cause gamma and low frequency components to decrease their coupling. However, previous studies have shown no effect of cueing on the characteristics of microssacades, such as shift, frequency, amplitude, or speed in spatial attention tasks (Mitchell et al., 2007). If switching attention toward the RF decreases low frequency power (Figure 1) by decreasing the link between microssacades and the low frequency component, the effect of attention on PAC should diminish when focusing the analyses on trials with an increase of low frequency power with attention. Instead we show that the effect of attention on PAC remains the same when selectively considering these trials (Supplementary Figure 6). These observations suggest that the modulation of PAC power is not caused by attention-related changes in microssacades.

Decorrelation of neurons has been reported as an effect of spatial attention in extrastriate visual cortex (Fries et al., 2001, 2008; Mitchell et al., 2009). Such an attentional effect supports better stimulus discrimination (Averbeck et al., 2006). Neuronal spike rates on the other hand are correlated with the power of high frequency LFPs in area MT (Liu and Newsome, 2006) and other extrastriate visual areas (Fries et al., 2008). Therefore, since in PAC the phase of low frequency oscillations determines the power of gamma, this phase also determines the spike rates of different neurons across time. Given that changes in PAC power would consequently reflect changes in the synchrony between different neurons' spiking activity, we propose that attention decorrelates neurons by decreasing the PAC power in extrastriate visual cortex. This can be addressed by investigating the link between PAC and coupling of spikes to the low frequency phase.

Our data show that the link between gamma power and spike rate decreases with attention during presentation of the same stimulus. This suggests indirectly that the link between gamma power and noise correlation decreases with attention. Furthermore, Womelsdorf et al. (2012) reported a negative correlation between gamma power and noise correlation in area V1. Considering that switching attention inside the RF increases gamma power (Figure 1), our observation confirms their finding.

The phase-power frequency pair with the highest PAC modulation differs across the individual animals in our study. Although the low frequency component of this pair (centered at 4 Hz) remains the same across the animals, the high frequency component shifts between the frequencies around 50 Hz (monkey T) and 90 Hz (monkey C). This suggests that attention modulates different high frequency components to control the synchrony of neurons. Similarly, Bosman et al. (2012) found that areas V1 and V4 of different monkeys become coherent at different gamma frequency bands during an attention task (Bosman et al., 2012).

In order to control for the effect of noise on the calculation of PAC, we carried out further analyses on site pairs with at least seventy five trials per attention condition. This revealed a patch of neighboring phase-power frequency pairs with significant increase in PAC power when attention was directed into the RF; The low frequency bins of the patch were centered at 7–9 Hz and the high frequency component at 70 Hz (data not shown). We also observed another patch of neighboring frequency pairs with a significant decrease in PAC power at the same frequency pairs as the main patch observed in the complete data of monkey H (Figure 3A). We further found a negative correlation between the PAC modulation at delta and theta bands across site pairs. This suggests that attending inside vs. outside of the RF shifts the low frequency component of PAC from delta to theta and low-alpha band. However since we did not have a sufficient number of trials for monkeys C and T, this analysis could only be carried out for one monkey.

In summary our data suggest that the phase of low frequency oscillations in extrastriate visual cortex, a signature of large scale neural activity, determines the power of high frequency oscillations, which reflect local information processing. Spatial attention modulates this coupling, presumably to decorrelate neurons and consequently increase the neural discrimination of visual stimuli.

## Author contributions

MD, ST designed research, MD performed research, ME analyzed data, ME, MD, ST wrote the paper.

### Conflict of interest statement

The authors declare that the research was conducted in the absence of any commercial or financial relationships that could be construed as a potential conflict of interest.
