# Supplementary material for: Attention Decreases Phase-Amplitude Coupling, Enhancing Stimulus Discriminability in Cortical Area MT
Source: Front Neural Circuits. 2015 Dec 22;9:82. doi: 10.3389/fncir.2015.00082 (PMC4686998; doi:10.3389/fncir.2015.00082)
Supplement: Supplementary file 1 [file Image1.pdf]

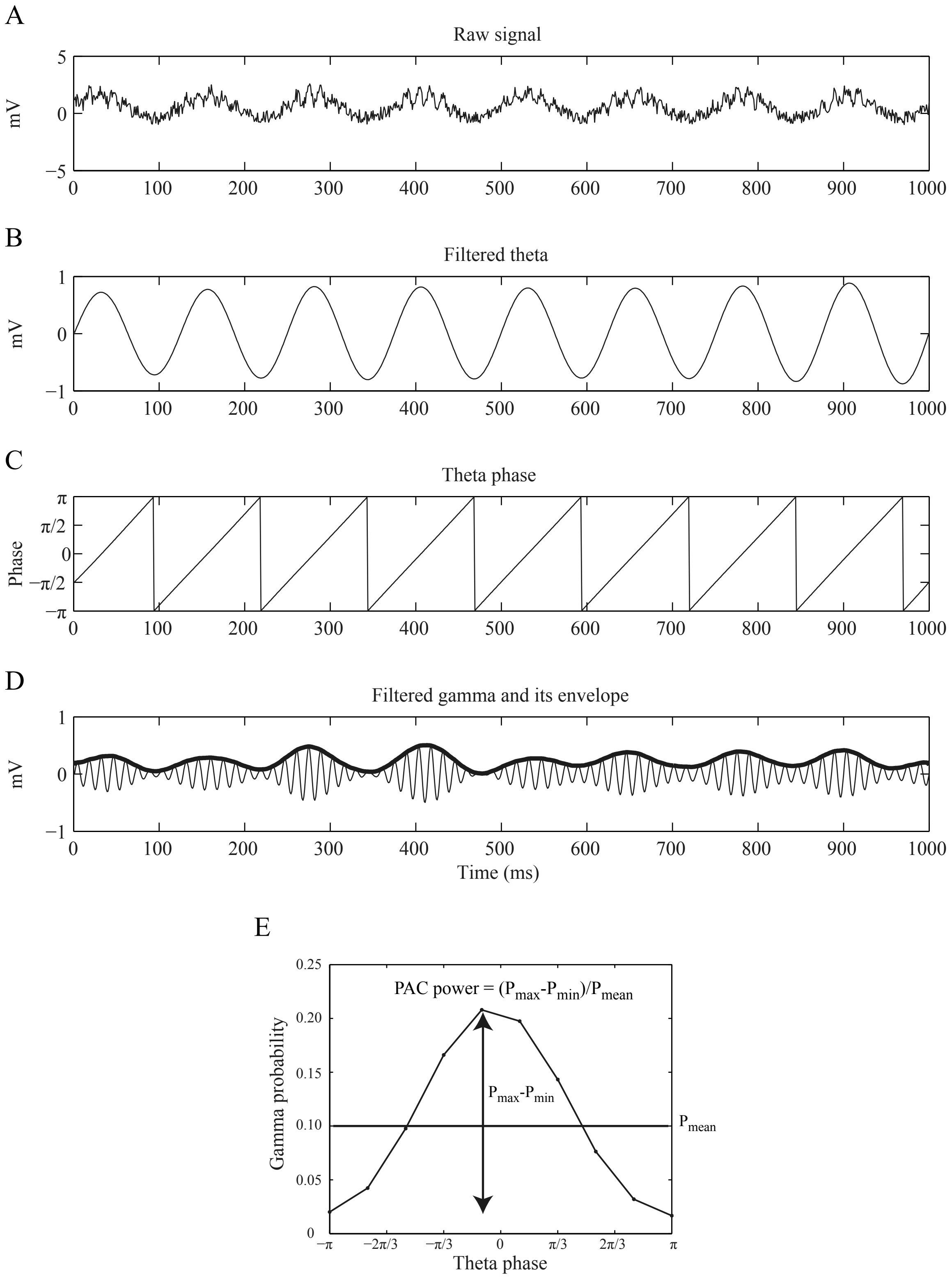

Supplementary Figure 1: Calculation of phase amplitude coupling (PAC). The figure illustrates the procedure of calculating PAC for an artificial signal consisting of an 8 Hz sinusoid (to represent theta) added to a 70 Hz sinusoid (to represent gamma) with gamma power varying based on the phase of theta where the maximum power occures at peaks of the theta component. A white noise signal is added to the sum of these two signals to simulate ongoing neuronal noise. (A) the artificially generated signal where the 70 Hz component is coupled to the 8 Hz component. (B, C) The raw signal is filtered into the low frequency band under study (here 7-9 Hz) and next given to Hilbert transform to calculate the instantaneous phases. Phases are computed by calculating the angles corresponding to the analytic signal given by Hilbert transform. (D) To calculate instantaneous power of gamma, the raw signal is first filtered into the high frequency band under study (here 65-75 Hz); second, the envelope of the signal is calculated by computing the absolute of the analytic signal given by applying Hilbert transform on the signal (shown by the bold black curve). Finally the second power of the envelope is computed as the instantaneous gamma power. (E) The dependency of gamma power on the theta phase is measured by first calculating the probability distribution function (PDF) of gamma power relative to theta phase using 10 equal phase bins partitioning the phase range (-π, π). Next we subtract trough (P_{min}) from the peak (P_{max}) value of the PDF and normalize the result by the mean probability (Pmean) that is 0.1 for our analyses (corresponding to the number of phase bins we use to estimate the PDF) (Tort et al., 2010). (Figures similar to Tort et al., 2010's Figures 1 & 5)
