# Supplementary material for: Attention Decreases Phase-Amplitude Coupling, Enhancing Stimulus Discriminability in Cortical Area MT
Source: Front Neural Circuits. 2015 Dec 22;9:82. doi: 10.3389/fncir.2015.00082 (PMC4686998; doi:10.3389/fncir.2015.00082)
Supplement: Supplementary file 2 [file Image2.pdf]

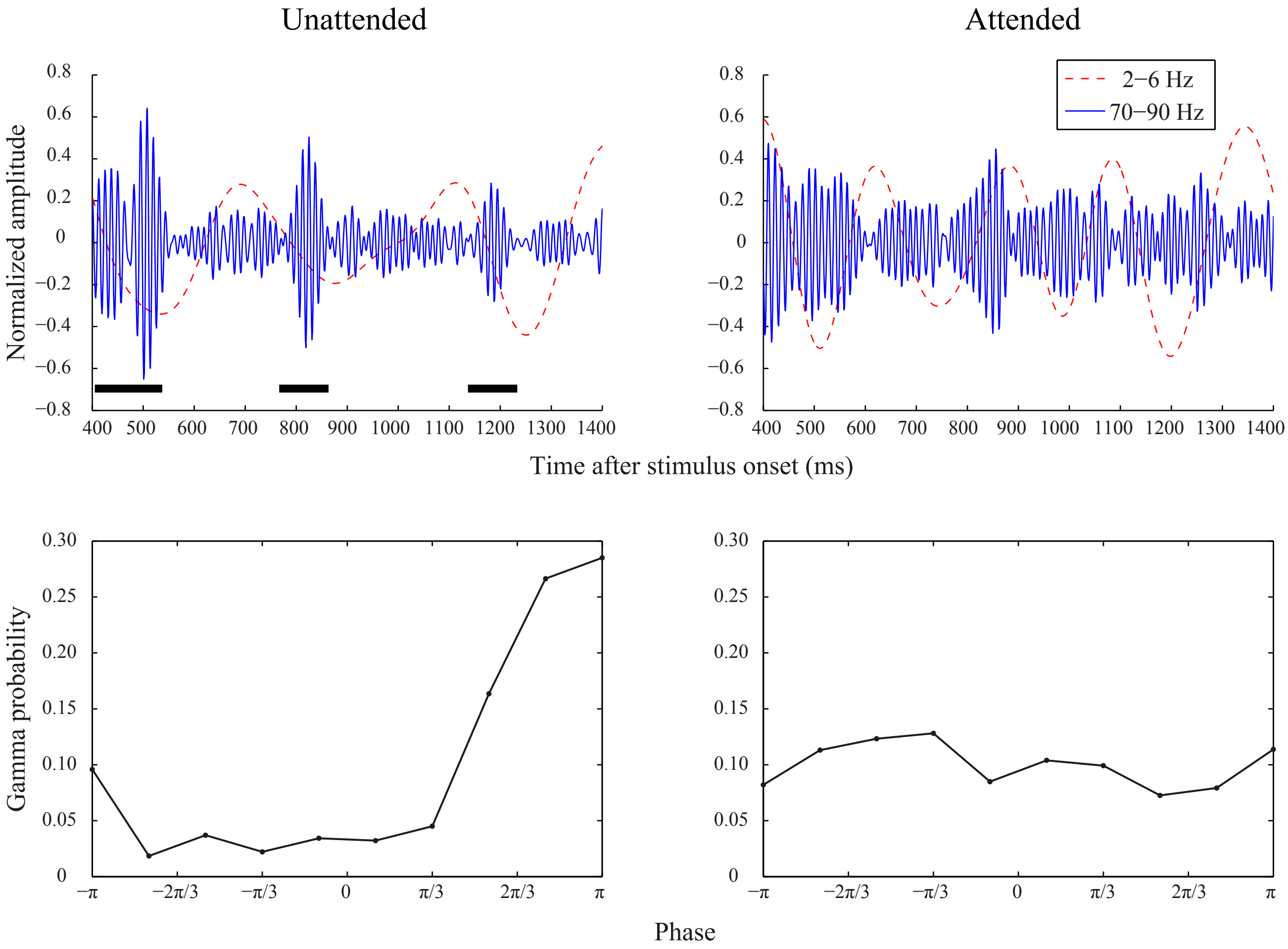

Supplementary Figure 2: Visualization of the locking between high frequency power and low frequency phase in a sample trial for each attention condition. Right and left panels each show a sample trial recorded from the same recording site of monkey H with the right and left one recorded when the monkey was attending inside or outside the RF, respectively. Figures in the top panel represent LFP filtered into two low (2-6 Hz) and high (70-90 Hz) frequencies shown as dashed and solid lines, respectively. Data are aligned to the onset of the stimuli on the screen. The bottom panels represent the probability distribution function (PDF) of the high frequency (gamma) power relative to the low frequency phase. PDFs are calculated using 10 equal phase bins dividing the phase range (-π, π). As shown in the top panel, power of gamma frequency is clearly higher during the falling phase of low frequency oscillations in the unattended trial (left panel) (indicated by solid lines below the curves), while there is no clear coupling between the two frequency bands in the attended condition (right panel). This is reflected in the PDFs (bottom panel) showing a larger peak-to-peak ampltiude difference in the unattended compared to the attended trial.
