# Supplementary material for: Attention Decreases Phase-Amplitude Coupling, Enhancing Stimulus Discriminability in Cortical Area MT
Source: Front Neural Circuits. 2015 Dec 22;9:82. doi: 10.3389/fncir.2015.00082 (PMC4686998; doi:10.3389/fncir.2015.00082)
Supplement: Supplementary file 3 [file Image3.pdf]

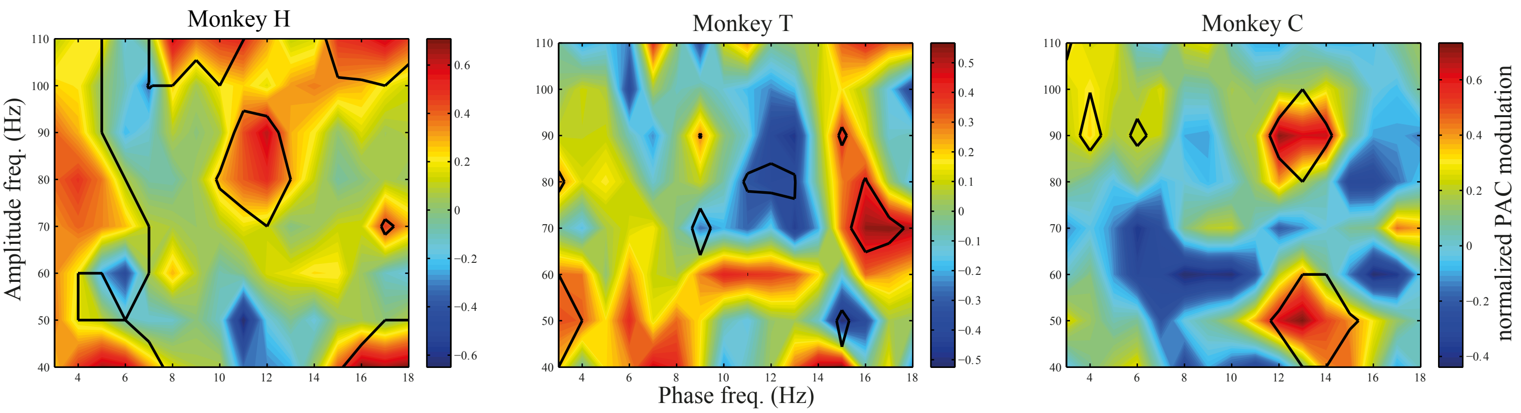

Supplementary Figure 3: Normalized PAC modulation map for monkeys H, C & T. The plots show PAC modulation after subtracting the peak-to-peak amplitude for the attended condition from the peak-to-peak amplitude for the unattended condition normalized to the mean peak-to-peak amplitude. Black lines indicate frequency pairs with significant PAC modulation ($p<0.05$ permutation test; corrected for multiple comparisons).
