# Supplementary material for: Attention Decreases Phase-Amplitude Coupling, Enhancing Stimulus Discriminability in Cortical Area MT
Source: Front Neural Circuits. 2015 Dec 22;9:82. doi: 10.3389/fncir.2015.00082 (PMC4686998; doi:10.3389/fncir.2015.00082)
Supplement: Supplementary file 4 [file Image4.pdf]

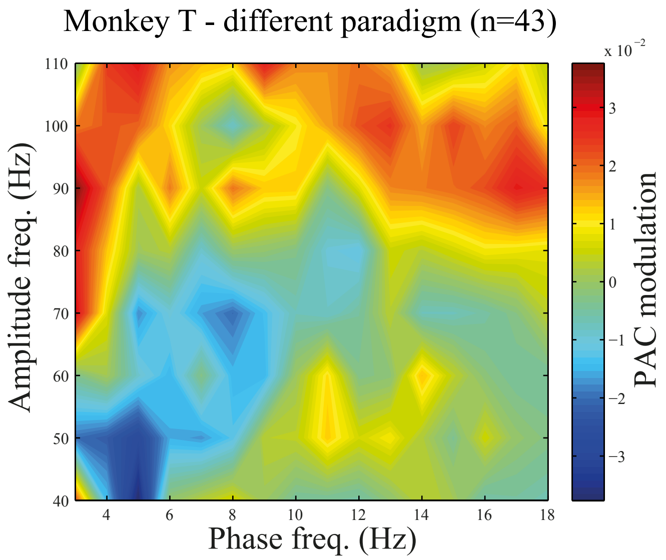

Supplementary Figure 4: PAC modulation map for monkey T for a spatial attention paradigm where the monkey had to detect a direction/color change in one of two moving RDPs based on the cue shown at the start of each trial. Due to large variations in the number of trials across recording sites in this dataset, each site's PAC is weighted by its number of trials. Details of the task are provided elsewhere (Katzner et al., 2009)
