# Supplementary material for: Attention Decreases Phase-Amplitude Coupling, Enhancing Stimulus Discriminability in Cortical Area MT
Source: Front Neural Circuits. 2015 Dec 22;9:82. doi: 10.3389/fncir.2015.00082 (PMC4686998; doi:10.3389/fncir.2015.00082)
Supplement: Supplementary file 5 [file Image5.pdf]

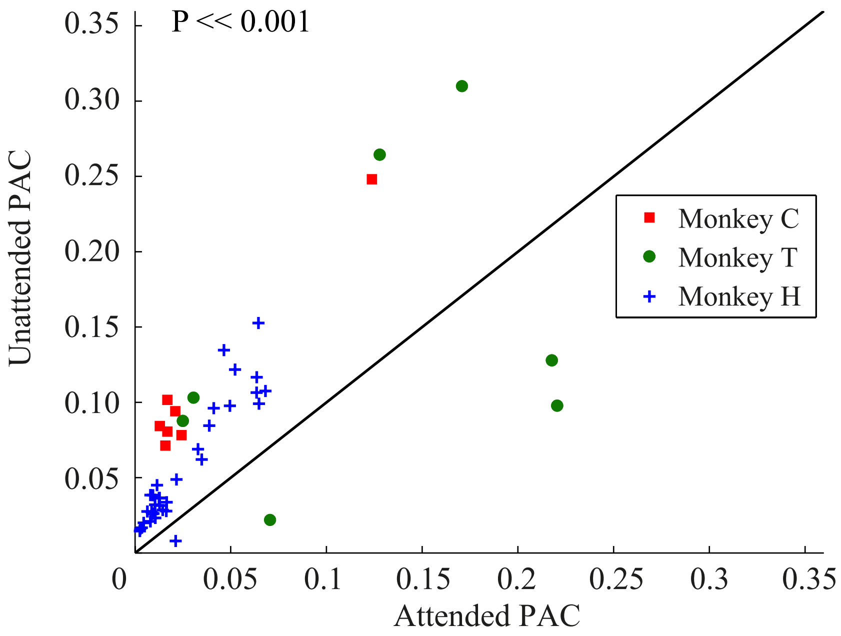

Supplementary Figure 5: PAC power for frequency pairs with significant modulation of PAC with attention. These pairs are shown in Figure 2 (A, C, E). Frequency pairs with higher PAC in the unattended condition outnumber frequency pairs with higher PAC in the attended condition for each monkey (P<<0.001 sign test, across frequency pairs from all monkeys).
