# Supplementary material for: Attention Decreases Phase-Amplitude Coupling, Enhancing Stimulus Discriminability in Cortical Area MT
Source: Front Neural Circuits. 2015 Dec 22;9:82. doi: 10.3389/fncir.2015.00082 (PMC4686998; doi:10.3389/fncir.2015.00082)
Supplement: Supplementary file 6 [file Image6.pdf]

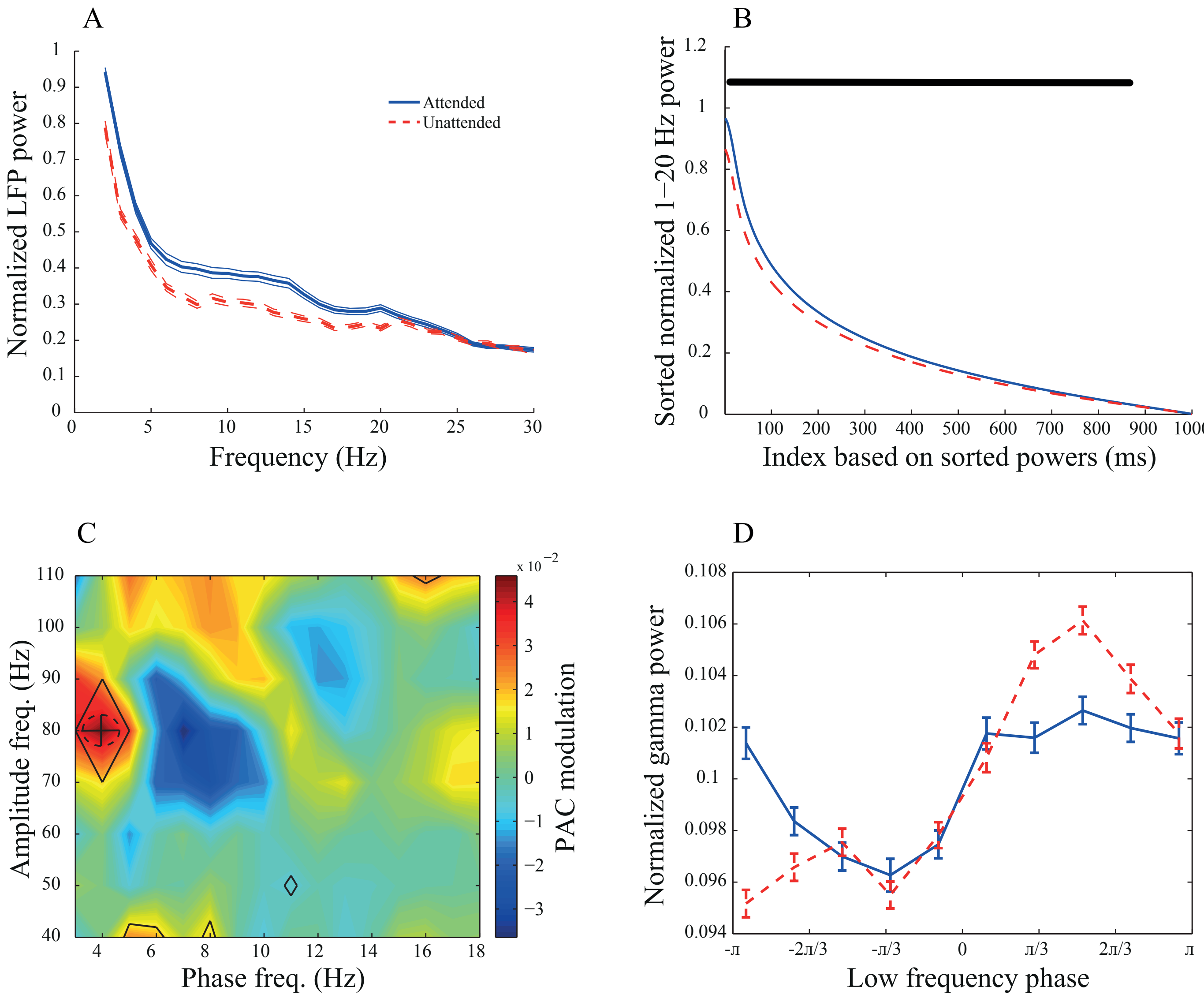

Supplementary Figure 6: Power-dependent differences in phase estimation accuracy do not explain attentional modulation of PAC. Here we selected a subset of trials (recorded from monkey H) in each attention condition such that attention is linked to an increase rather than decrease of power in low frequencies in this subset. For each site we selected those attended and unattended trials, where the average 1-20 Hz power was higher and lower, respectively compared to the average 1-20 Hz power of the site. Panel A shows the average power spectral density for each attention condition based on the trial selection. To ensure that trials selected using this method do not differ in their low frequency power across the attention conditions only in some parts of each trial, we sorted the 1-20 Hz power across time for each of the trials selected in the previous step. Instantaneous powers were quantified by calculating the envelope of the filtered signals (Figure S1D). Panel B reflects the sorted powers averaged over recorded sites. The solid line shows indices with a significant difference between the two attention conditions ($p<0.01$, sign test, corrected for multiple comparisons). This indicates that to the extent of the selected trials, attention is linked to an increase of low frequency power uniformly through each trial. Panel C shows the PAC modulation map similar to Figure 2A based on the selected set of trials. Panel D shows the PDFs for each attention condition at the frequency pair with maximum PAC modulation in Figure 2A (2-6 Hz, 70-90 Hz) which also corresponds to the maximum PAC modulation in panel C. The PDFs show that the attended condition has a smaller peak-to-peak amplitude compared to the unattended condition ($p<<0.01$, permutation test). This indicates that the attentional suppression of PAC cannot be explained by power-dependent differences in phase estimation accuracy.
